# Cutaneous Melanoma Classification: The Importance of High-Throughput Genomic Technologies

**DOI:** 10.3389/fonc.2021.635488

**Published:** 2021-05-28

**Authors:** Cristian Scatena, Daniela Murtas, Sara Tomei

**Affiliations:** ^1^ Division of Pathology, Department of Translational Research and New Technologies in Medicine and Surgery, University of Pisa, Pisa, Italy; ^2^ Department of Biomedical Sciences, Section of Cytomorphology, University of Cagliari, Cagliari, Italy; ^3^ Omics Core, Integrated Genomics Services, Research Department, Sidra Medicine, Doha, Qatar

**Keywords:** melanoma, genomics, next-generation sequencing, DNA, mutations

## Abstract

Cutaneous melanoma is an aggressive tumor responsible for 90% of mortality related to skin cancer. In the recent years, the discovery of driving mutations in melanoma has led to better treatment approaches. The last decade has seen a genomic revolution in the field of cancer. Such genomic revolution has led to the production of an unprecedented mole of data. High-throughput genomic technologies have facilitated the genomic, transcriptomic and epigenomic profiling of several cancers, including melanoma. Nevertheless, there are a number of newer genomic technologies that have not yet been employed in large studies. In this article we describe the current classification of cutaneous melanoma, we review the current knowledge of the main genetic alterations of cutaneous melanoma and their related impact on targeted therapies, and we describe the most recent high-throughput genomic technologies, highlighting their advantages and disadvantages. We hope that the current review will also help scientists to identify the most suitable technology to address melanoma-related relevant questions. The translation of this knowledge and all actual advancements into the clinical practice will be helpful in better defining the different molecular subsets of melanoma patients and provide new tools to address relevant questions on disease management. Genomic technologies might indeed allow to better predict the biological - and, subsequently, clinical - behavior for each subset of melanoma patients as well as to even identify all molecular changes in tumor cell populations during disease evolution toward a real achievement of a personalized medicine.

## Introduction on Cutaneous Melanoma

Cutaneous melanoma represents an aggressive tumor with a continuous increase in incidence, although mortality rates have begun to decline thanks to promising new targeted treatments (1). The incidence of cutaneous melanoma is increasing in white populations worldwide, in particular if people receive excessive sun exposure (2–4). In the United States the incidence is 20-30 cases per 100,000 inhabitants, while in Australia it is particularly high, with a rate of 50-60 cases per 100,000 inhabitants. In Europe, instead, the incidence is <10-25 cases per 100,000 inhabitants (5), but it has been predicted to increase in the next decades (6).

Factors that increase the risk for melanoma include: *i)* fair skin, that easily burns in the sun; *ii)* the presence of numerous common naevi, large congenital naevi or atypical (dysplastic) naevi, commonly genetically determined (7, 8); *iii)* exposure to UV irradiation, in particular high and intermittent sun exposure (9); *iv)* genetic susceptibility, as inherited variants of melanocortin-1 receptor (MC1R); *v)* a family history of melanoma.

**Figure 1:**
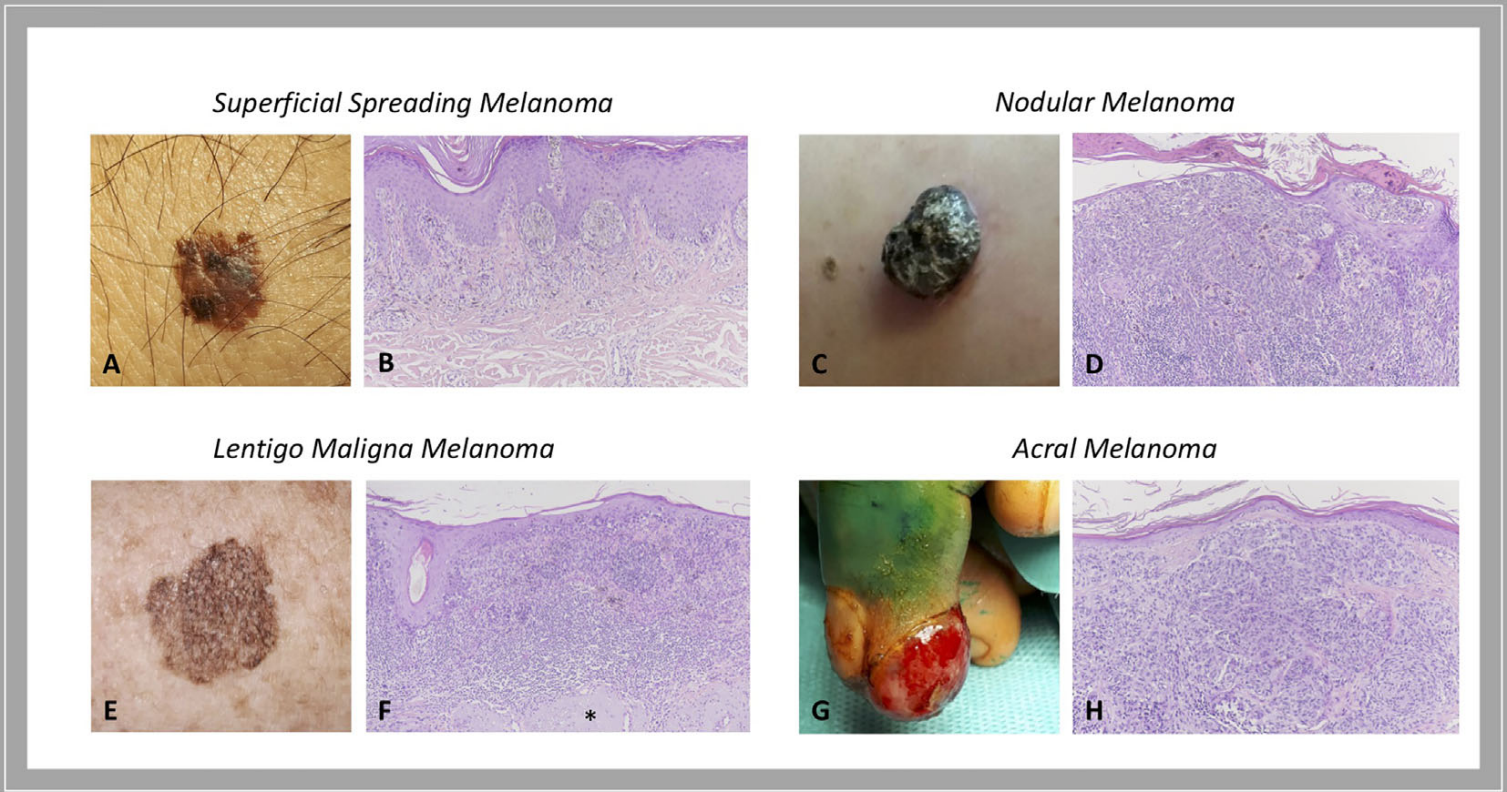
Histological subtypes of melanoma: clinical-pathological correlations. Superficial spreading melanoma is a pigmented macule with irregular contours **(A)** that, when invasion occurs, presents as a proliferation of atypical melanocytes in the papillary dermis **(B)**. Nodular melanoma is a brown-to-black exophytic tumor **(C)** characterized by a predominant vertical growth phase, with pigmented epithelioid or spindle atypical melanocytes that invade the reticular dermis **(D)**. Lentigo maligna melanoma presents as a large, pigmented macule with irregular contours on sun damaged skin **(E)** that is described histologically as a lentiginous proliferation of atypical spindle melanocytes at the dermo-epidermal junction with invasion into the papillary dermis; actinic damage and dermal elastosis (*) are typically present in the surrounding skin **(F)**. Acral melanoma may be an amelanotic nodule localized on the extremities **(G)** characterized by a proliferation of atypical, not pigmented, spindle melanocytes throughout the dermis **(H)**. Courtesy of Dermatology Unit, Department of Clinical and Experimental Medicine, University of Pisa.

As for most tumors, also cutaneous melanoma is traditionally classified into primary and metastatic; primary melanoma is further divided into: i) melanoma *in situ*, when the atypical melanocytes are limited to the epidermis; ii) invasive melanoma, if it conquers the dermis. Invasive melanoma is historically classified according to clinical and histopathological characteristics into four major histological subtypes: *i)* superficial spreading melanoma (SSM), which accounts for 41% of cases; *ii)* nodular melanoma (NM), accounting for 16% of cases; *iii)* lentigo maligna melanoma (LMM), accounting for 2.7% - 14% of cases; and *iv)* acral melanoma (AM), accounting for 1% - 5%, with acral lentiginous melanoma (ALM) that represents its most common subtype (**Figure 1**). For the latter subtype, higher rates are reported in Asian and African American population (10, 11). In details:

- *In situ*/SSM appears as a pigmented macule with irregular contours that may progressively evolve into a papule or plaque, so far as invasion occurs; histologically, melanoma *in situ* is defined as the presence of a pagetoid spread of malignant melanocytes throughout the epidermis; instead, invasive SSM presents as a proliferation of atypical melanocytes in the superficial dermis.
- NM appears as an exophytic/nodular, brown-to-black, often eroded tumor, characterized by a vertical growth phase. The epidermal lateral component, when present, is observed within three rete ridges, at the maximum.
- LMM represents the invasive progression of melanoma *in situ*/lentigo maligna and is mainly located on the sun damaged surfaces, as the face of elderly people (12). Histologically, lentigo maligna is described as a lentiginous proliferation of atypical spindle melanocytes along the base of the epidermis, without invading the dermis; instead, LMM has at least single cell infiltration into the papillary dermis. Actinic damage and dermal elastosis are typically present in the surrounding skin.
- To conclude, AM is a slow-growing macule/plaque or nodule localized on the extremities (subungual or palmoplantar/volar skin) with poorly circumscribed pigmentation. The most frequent histological subtype is ALM, followed by NM and SSM. A proliferation of atypical spindle (often pigmented) melanocytes at the base of the epidermis constitutes ALM. Rarely AM manifests as a large amelanotic nodule that easily can be misdiagnosed as a benign condition.

Rare histological subtypes of cutaneous melanoma are: *v)* desmoplastic melanoma (DM) (1% - 4% of cases) (13); and *vi)* amelanotic melanoma (14).

Curiously, in the current staging system for cutaneous melanoma (American Joint Committee on Cancer – AJCC, 8th edition, 2017) histological subtypes are not mentioned as prognostic factors (15). Instead, important markers of worse prognosis include: i) vertical tumor thickness (Breslow’s depth); ii) ulceration; iii) number of mitosis/mm^2^ (no longer used for sub-classification); iv) deepness of invasion (Clark’s level); v) tumor infiltrating lymphocytes (TILs); vi) lymphovascular invasion; and vii) neurotropism. Also, older age, the male sex and the localization to head and neck or trunk are associated to a poorer outcome (16, 17). However, the identification of genetic alterations in specific subtypes of cutaneous melanoma has made histological classification regain prominence (18). Indeed, in the latest WHO classification of skin tumors (4th edition, 2018), melanoma is classified according to the association with sun-exposure and genomic features. Melanomas that arise in sun-exposed skin include: i) melanoma in skin with a low degree of cumulative sun damage (low-CSD melanoma), mostly SSM; ii) melanoma in chronically sun-exposed skin, mostly LMM and desmoplastic melanoma. NM may belong to both categories. Instead, Spitz melanoma, melanoma developed in congenital or blue naevus, acral melanoma, melanoma arising in blue naevus, mucosal melanoma (oral, genital or sinonasal), uveal melanoma, nevoid and some nodular melanomas arise in sun-sheltered sites (11).

Instead, metastatic melanoma is defined as a melanoma that has spread to other sites of the body. Melanoma may metastasize locally through the lymphatic system (as satellite, in-transit, or regional nodal metastases) or systemically through the hematic route to distant skin/subcutaneous tissue or lymph nodes, lung, liver or brain (19).

## Current Knowledge of Genetic Alterations in Cutaneous Melanoma

The initiation and progression of cutaneous melanoma are finely driven by specific genomic alterations (20, 21). Although hundreds of genes can be found mutated in a single case of cutaneous melanoma, only some mutations are true “drivers” of the tumor, either as gain-of-function (GOF)/activating or loss-of-function (LOF)/deleterious mutations. Melanoma may display mutations in known oncogenes that then result overactive in melanoma cells, granting uncontrolled tumor growth. Mutations may also occur in tumor suppressor genes that control cell growth; when mutated, those genes lose their function. Their inactivation may thus result in the activation of downstream growth pathways, allowing unchecked tumor growth (22–24).

In the last decades, the driving alterations leading to cutaneous melanoma have been largely catalogued, comprising both activating and deleterious mutations, and including single nucleotide variants (SNVs, somatic and germline mutations) and copy number variations (CNVs). Somatic mutations are genetic alterations occurring in single cells of somatic tissues. When mutated, such cells undergo uncontrolled division and can be causative of melanoma. Germline mutations are less common and occur within melanoma-predisposing genes in the germ line, thus they can be passed on from one generation to the next, leading to the so-called hereditary or familial melanomas (25).

The current knowledge on genetic alterations is catalogued and continuously updated in databases such as The Skin Cutaneous Melanoma catalogue in The Cancer Genome Atlas (TCGA), Pan-Cancer Atlas data set (available at: https://cancergenome.nih.gov), the cBioPortal for Cancer Genomics (available at: www.cbioportal.org), OncoKB, ClinVar, “1000 Genomes” project, and Cancer Hotspots (available at: www.cancerhotspots.org) (26, 27), as described later in this review.

Activating mutations occur in oncogenes. The two most frequent alterations, commonly mutually exclusive both in cell lines and tumors, have been described in the kinase domain of B-Raf Proto-Oncogene, Serine/Threonine Kinase (*BRAF*), encoded by exons 11 and 15, and in exons 2, 3 and 4 of Neuroblastoma RAS Viral Oncogene Homolog *(NRAS)* gene, with a frequency of 50-70% and 15-30%, respectively (20, 21, 27–31).

*BRAF* encodes for a serine/threonine protein kinase of the Rapidly Accelerated Fibrosarcoma (RAF) family, which transfers growth signals to the cells, playing a pivotal role in activating the mitogen-activated protein kinase/extracellular signal-regulated kinase (MAPK/ERK) signaling pathway and influencing cell cycle, differentiation, and apoptosis. More than 90% of *BRAF* gene mutations occur at codon 600 of exon 15, within the activation segment of the kinase, by substitution of a single nucleotide (GTG to GAG), which results in a single amino acid substitution from valine (V) to glutamic acid (E) (BRAF-V600E). The BRAF^V600E^ mutation has been described to confer a 400-fold increased activity to the protein (20, 31). Another prevalent *BRAF* mutation at the same residue, accounting for 10-30% of all BRAF^V600^-mutated melanomas, is V600K mutation (BRAF-V600K) in which the valine residue (V) is replaced by a lysine (K) through a two nucleotides substitution (GTG to AAG) (32). A small proportion, about 1-5% of melanoma patients, harbor mutations at codon K601 in exon 15 of the *BRAF* gene (BRAF-K601E), the third most common type of *BRAF* mutation, resulting in a single amino acid change from lysine (K) to glutamic acid (E) (33–35). *BRAF* mutation and expression have also been shown to affect the immunological phenotype of melanoma. By functional interpretation analysis of 6296 genes differentially expressed between BRAF-mutant samples with high or low BRAF mRNA expression, Interleukin 2 (IL-2) and Janus Kinase/Signal Transducers and Activators of Transcription (JAK/STAT) signaling emerged among the deregulated pathways, supporting the immunoregulatory role of BRAF in melanoma (21).

*NRAS* oncogene is a member of the superfamily of p21 GTPases, which have intrinsic GTPase activity, playing as a molecular switch for the transmission of regulatory cell signals. These proteins participate in the activation of the MAPK/Phosphoinositide-3-Kinase (MAPK/PI3K) pathway, during cell proliferation, differentiation, and survival.

Although *NRAS* mutations associated with malignant transformation have been predominantly detected in codons 12, 13 (exon 2), and 61 (exon 3), the most common *NRAS* gene mutation in cutaneous melanoma occurs at position 61, where glutamine (Q) is substituted by arginine (R), lysine (K), or leucine (L) (NRAS-Q61R/K/L). *NRAS* mutations lead to the reduction of the intrinsic GTPase activity of NRAS and its constitutive activation, with consequent growth factor-independent melanocyte proliferation and ultimately melanomagenesis (20, 30, 31, 36).

Additionally, high-frequency activating mutations have been identified in Ras-related C3 Botulinum Toxin Substrate 1 (*RAC1*), Mast/Stem Cell Growth Factor Receptor Kit (*KIT*), Telomerase Reverse Transcriptase (*TERT*) promoter region (*TERT*prom), Mitogen-Activated Protein Kinase Kinase 1 and 2 (*MAP2K1* and *MAP2K2*), G Protein Subunit Alpha Q (*GNAQ*), G-Protein Subunit α11 (*GNA11*), Isocitrate Dehydrogenase 1 (*IDH1*), Erb-b2 Receptor Tyrosine Kinase 2/4 (ERBB2/4), Kirsten Rat Sarcoma Viral Oncogene Homolog, GTPase (*KRAS*), and Splicing Factor 3b Subunit 1 (*SF3B1*) genes (24, 27, 37–42).

A recurrent activating mutation in *RAC1*, a RAS-related member of the Rho GTPases subfamily, has been identified in 9.2% of sun-exposed melanomas. This C>T transition (CCT to TCT) results in a proline (P) to serine (S) amino acid substitution and it has been described as consistent with a molecular signature associated with UV radiation damage. The RAC1 P29S mutation is more frequent in melanomas BRAF and NRAS wild-type and occurs early in tumorigenesis. Activated mutant RAC1 shows enhanced binding activity towards RAC1 downstream effectors and its expression leads to increased melanocyte proliferation, altered cell migration, and activated MAPK signaling (38, 43, 44).

The tyrosine-protein kinase Kit acts as a cell surface receptor regulating proliferation and survival, by activating the MAPK, PI3K, and JAK/STAT pathways. *KIT* (C-KIT/CD117) gene mutations show heterogeneous distribution through the gene and they have been detected in hot-spots at exon 9 (c459/465/471/483), 11 (c551/559/576), 13 (c642), and 17 (c816), accounting for 5-15% of mutations of diagnosed melanomas. In light of the relatively high mutation rate of *KIT* in cutaneous melanoma and since *BRAF*, *KIT*, and *NRAS* mutations appear to be mutually exclusive, the screening of *KIT* mutations, at least in exons 9/11/13, is suggested in BRAF/NRAS double-wild-type melanoma patients (31, 34, 45, 46).

The *TERT* gene encodes the catalytic subunit of telomerase, responsible for the maintenance of chromosomal telomere length, thus sustaining cell survival. Mutations in the *TERT*prom lead to a 2-fold to 4-fold increase in the transcription of *TERT*, along with enhanced telomerase activity, and are often found in BRAF^V600^ and NRAS-mutant melanomas, where the combined alterations cooperate in boosting cancer progression and aggressiveness. The two most recurrent, mutually exclusive, *TERT*prom mutations are cysteine (C) to threonine (T) mutations located at position 228 (C228T) and 250 (C250T) (34, 37, 47).

Deleterious mutations in tumor suppressor genes most frequently affect Neurofibromin 1 (*NF1*), Phosphatase and Tensin Homolog (*PTEN*), Tumor Protein 53 *(TP53)*, RAS P21 Protein Activator 2 (*RASA2)*, Protein Phosphatase 6 Catalytic Subunit (*PPP6C*), and genes encoding SWItch/Sucrose Non-Fermentable (SWI/SNF) subunits, most commonly AT-Rich Interaction Domain 2 (*ARID2*) (23, 27, 48).

NF1 is a tumor suppressor protein that plays a pivotal role in the control of cell growth by negatively regulating Rat Sarcoma (RAS) proteins. The GTPase-activating protein (GAP)-related domain of NF1 is known to convert the active RAS-guanosine triphosphate (RAS-GTP) to the inactive RAS-guanosine diphosphate (RAS-GDP), thereby inhibiting downstream RAS signaling (49). The *NF1* gene is mutated in 10–15% of melanoma cases. By large-scale targeted sequencing, whole-exome sequencing (WES), and whole-genome sequencing (WGS), *NF1* has been established as one of the key drivers of melanoma. Most *NF1* mutations cause a loss-of-function of this tumor suppressor gene, with about 80% of patients having a nonsense mutation, an insertion, or a deletion that leads to a truncated protein. *NF1* loss−of−function induces the hyperactivation of NRAS protein and thus, the activation of MAPK and PI3K signaling pathways (50). These *NF1* mutations are more common in melanomas occurring on chronically sun-exposed skin or in older patients, in melanomas with higher mutation burden, wild-type for *BRAF* and *NRAS*, and in the desmoplastic melanoma subtype (28, 49, 51).

*PTEN* is a well characterized tumor suppressor gene that encodes for the PTEN protein, a key negative regulator of the PI3K signaling pathway and an effector of apoptosis through Protein Kinase B/AKT Serine/Threonine Kinase (PKB/AKT). Somatic *PTEN* alterations have been identified in 14% of cases in the TCGA melanoma cohort, comprising both mutations and focal deletions. *PTEN* mutations frequently coexist with *BRAF* mutations, but not with *NRAS* ones. Reportedly, *PTEN* loss in melanoma is a frequent event, occurring in about 30% of primary tumors, with an even higher frequency in melanoma cell lines (47, 52). The loss of functional PTEN leads to reduced apoptosis along with increased mitogen signaling and cell survival, thus promoting tumor progression (53). Moreover, PTEN loss can influence the immune microenvironment in terms of a poor T- and B-cell tumor infiltration, sustaining immune evasion (54). T cell-based immunotherapy approaches have shown promising results in melanoma (55, 56). Yet, some patients do not respond to these therapeutic approaches. The loss of PTEN has been reported to be a molecular determinant that might explain immune resistance due to its inhibition of the T cell trafficking into tumors (57).

**Figure 2:**
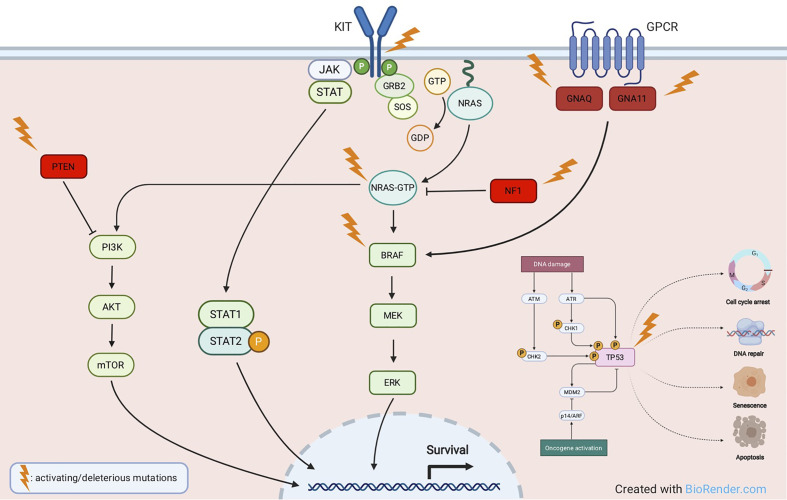
Main molecular pathways involved in melanomagenesis. Activating mutations are commonly detected in oncogenes like *KIT, NRAS, BRAF, GNAQ, GNA11*, whereas deleterious mutations most frequently affect tumor suppressor genes like *NF1, PTEN* and *TP53*.

The *TP53* gene is considered the “guardian of the genome” due to its pleiotropic function in protecting cells from genotoxic damages, acting as tumor suppressor and transcriptional activator/repressor of several downstream genes controlling cell-cycle progression, DNA repair, and also triggering apoptosis (58, 59). *TP53* mutations have been reported in about 15% of TCGA cases, they are mostly ultraviolet (UV) radiation-induced, and lead to tumor initiation and progression. In melanoma, p53 wild-type form may get inactivated by a variety of mechanisms, including inactivation of p14 which in turn causes overexpression of the Mouse Double Minute 2 (*MDM2)* proto-oncogene (48, 60). *TP53* is mutated in melanomas harboring any of the major subsets of *BRAF*, *NRAS*, or *NF1* mutations. Conversely, in triple-wild-type tumors, there is a prevalent amplification of *MDM2*, a key regulator of p53 protein that ubiquitinates p53, leading to its degradation (50). Loss-of-function of mutated *TP53* causes a critical dysregulation of diverse apoptotic pathways, supervised by p53, including Caspase3, Fas Cell Surface Death Receptor (FAS), and cytotoxic T-cell (CTL)-mediated apoptosis. Moreover, inactivity of mutant *TP53* decreases the surface level of the major histocompatibility complex (MHC)-peptide complex, resulting in downregulated immune surveillance (61). **Figure 2** illustrates the main molecular pathways involved in melanomagenesis.

Specific classes of cutaneous melanoma have been associated to specific genetic alterations. In particular: i) low-CSD melanoma (located on the trunk or extremities and belonging to the superficial spreading or nodular histological subtypes) carries *BRAF* mutations; ii) melanoma in chronically sun-exposed skin (located in the head and neck region) carries *NRAS* and/or other *RAS* mutations; iii) non sun-related melanomas (located on acral sites or mucosae) carry *C-KIT* mutations or amplifications (62).

**Figure 3:**
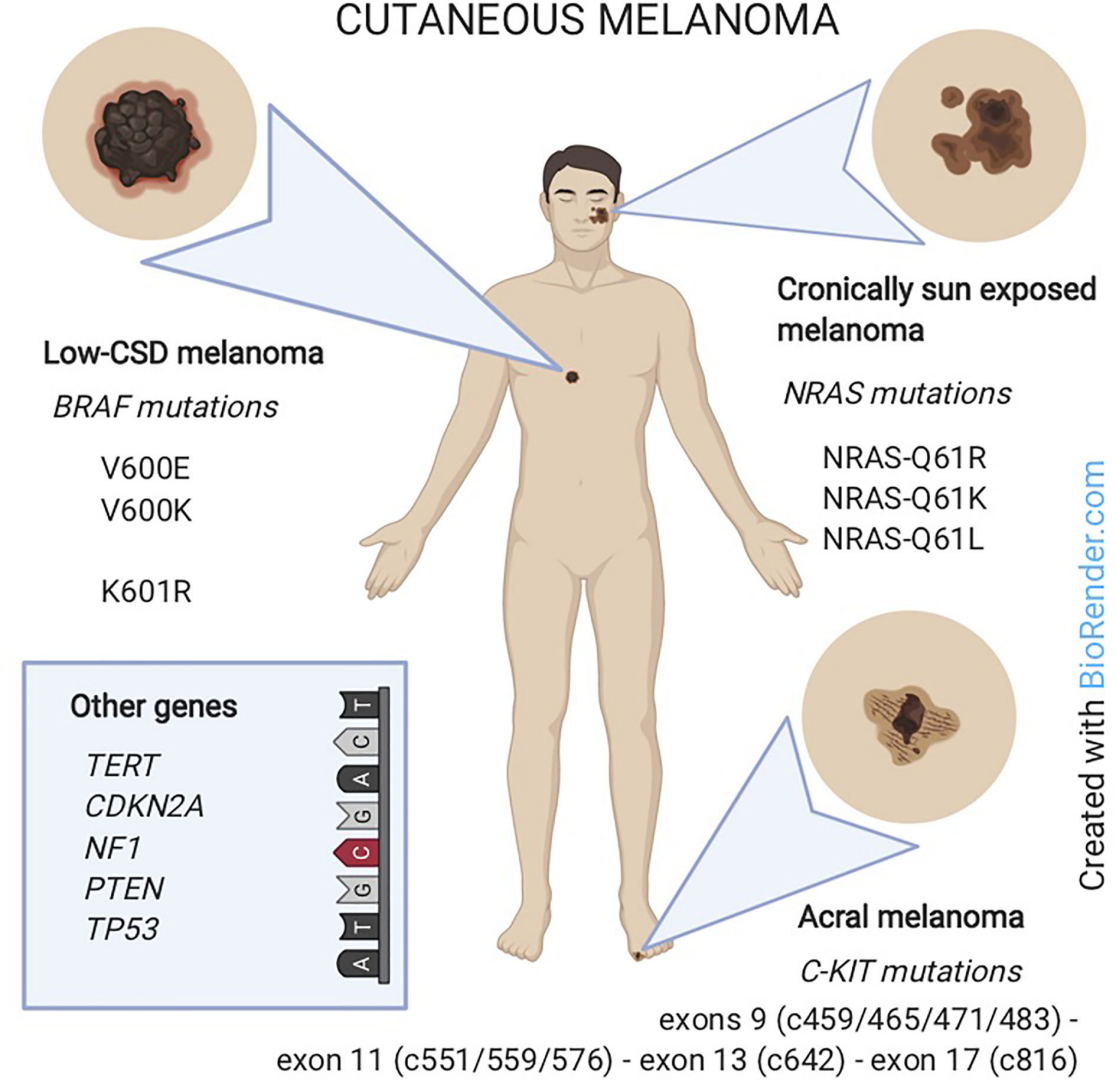
Melanoma classification according to the association with sun-exposure and genomic features. Low-CSD melanoma (mainly on the trunk) carries *BRAF* mutations. Melanoma in chronically sun-exposed skin (in the head and neck region) carries *NRAS* mutations. Non-sun-related melanomas (on acral sites) carry C-KIT mutations. In rare cases, other genes as *TERT*, *CDKN2A*, *NF1*, *PTEN* or *TP53* are involved.

Moreover, *BRAF*-mutated melanomas are more common in younger patients (63) whereas NRAS mutations are encountered in older patients and in the nodular histological subtype (64). On the other hand, most AM do not display mutations in *BRAF* or *NRAS* but bear *C-KIT* alterations (SNVs or amplifications) in 3-36% of cases (65) (**Figure 3**).

The genomic profiling of cutaneous melanoma represents a great tool to improve the management of patients with such an aggressive disease since it carries the potential to increase prognostic accuracy and to promote the development and optimize the use of molecular targeted therapies (66). The identification of genomic alterations through genomic analysis (such as DNA sequencing) is expected to promote the tuning of novel, fast and easy-to-use tests for patients’ stratifications (67).

About 90% of melanomas are primary tumors without metastatic dissemination. For such diseases the tumor-specific 10-year-survival is about 75-95%. Interestingly, the relationship between survival and tumor driven mutational status has been extensively investigated: *BRAF*-mutated melanoma has been associated with a shorter survival in patients with both metastatic (68, 69) and early-stage disease (70, 71); moreover, for patients with metastatic *BRAF*-mutated melanoma receiving BRAF inhibitors, a worse prognosis has been also associated with alterations in the thrombophilic status, such as high D-dimer levels at baseline (72, 73). *NRAS* mutations did not display any effect on the survival if measured in the primary tumor (74, 75); instead, if measured in the metastases, *NRAS* mutations were associated with improved survival (76, 77). On the other hand, in *NRAS*-mutated melanoma data on survival result conflicting: some studies report no difference in patients’ survival (74, 75), whereas in one study *NRAS* mutations were associated with improved survival in metastatic disease (78, 79).

According to the TCGA Pan-Cancer Atlas data set, 65% of melanomas that have *BRAF*, *NRAS*, *NF1*, or *KIT* as driver mutation co-occur with mutations in at least one other pathway, most frequently affecting *PTEN*, Cyclin-Dependent Kinase Inhibitor 2A (*CDKN2A*), and *TP53* (27, 50).

Regarding somatic CNVs assessment, deletions have been identified most frequently in the tumor suppressors PTEN, *PPP6C*, and *CDKN2A* genes, while amplifications occur repeatedly in *KIT*, Epidermal Growth Factor Receptor *(EGFR)*, and Cyclin-Dependent Kinase 4 (*CDK4*) oncogenes. CNVs influence particularly the CDK4 pathway, as also suggested by the fact that *CDKN2A* deletion or *CDK4* amplification result in CDK4 pathway activation. This pathway results altered in more than 40% of metastatic melanoma patients, including the majority of those with *NRAS*-mutant tumors (27). In a recent study, Melanocyte Inducing Transcription Factor (*MITF*) and *EGFR* genes have shown the highest frequency of genomic amplification, with a lower rate in primary melanomas as compared to metastatic melanomas, considering both tumor tissues and cell lines (80).

The diagnosis of primary melanoma is not always straightforward, especially when histologic features of the lesion overlap with those of various precursor lesions. Moreover, occasionally, melanomas can either lose their antigenicity to melanocytic markers or even show aberrant expression of non-melanocytic markers. The diagnostic uncertainty can thus lead to significant therapeutic implications (81). For this reason, the mutational testing could contribute to a more accurate diagnosis (82, 83).

Large-scale sequencing projects cataloguing mutations in cutaneous melanoma have been carried out mostly on advanced melanomas, overlooking the time of occurrence of genetic changes during tumor progression. Cutaneous melanomas often arise from distinctive precursor lesions such as melanocytic naevi, intermediate lesions, or melanoma *in situ*. By next generation sequencing (NGS) and targeted sequencing techniques, with a panel of cancer-relevant genes, Shain et al. (84) have recently proposed an “evolution/progression model” uncovering the sequence of pathogenic mutations occurring from precursor to malignant melanocytic lesions, trying to define a genetic signature for each stage of the neoplastic progression. As melanoma progresses, the pattern of genetic changes leads to genetically distinct subpopulations, that account for tumor heterogeneity.

Early lesions show the BRAF^V600E^ mutation as the only apparent pathogenic mutation, implying that BRAF^V600E^ may occur early in naevi as a putative driving alteration. Lesions classified as intermediate by histopathological characteristics and melanomas *in situ* harbor a broader spectrum of oncogenic alterations, including BRAF^V600K^ or BRAF^K601E^, *NRAS*, *GNAQ* or *GNA11*, and *TERT*prom mutations, showing genetic differences between benign and malignant neoplasms. Copy-number alterations are common in descendant neoplasms. Loss of *CDKN2A*, as well as mutations in *ARID2* gene, emerge exclusively in invasive melanomas. Finally, *PTEN* and *TP53* alterations increase exclusively in advanced melanomas, implying that these mutations may occur later and contribute significantly to tumor progression. It seems thus clear that the tumor mutation burden increases from benign through intermediate lesions to melanoma (84).

Among melanocytic diseases, Spitz tumors include Spitz nevus, atypical Spitz tumor (AST) and Spitz melanoma (or Malignant Spitz Tumor, MST), a challenging diagnostic group. The genetic characterization of these lesions and the identification of novel molecular markers are useful to improve the differential diagnosis of such diseases, the prediction of their biological behavior, and the achievement of efficient personalized treatments. The mutations driving the growth of benign Spitz naevi, considered initiating alterations, include Harvey Rat Sarcoma Viral Oncogene Homolog (*HRAS)* mutations, most frequently Q61K/R in exon 3, BRAF^V600E^, as well as larger genomic rearrangements involving the Anaplastic Lymphoma Receptor Tyrosine Kinase (*ALK*), Neurotrophic Receptor Tyrosine Kinase 1 (*NTRK1*), Ret Proto-Oncogene (*RET*), ROS Proto-Oncogene 1, Receptor Tyrosine Kinase (*ROS1*), Met receptor tyrosine kinases (*MET RTK*s), and *BRAF* genes. The pathogenesis of AST mostly derives from mutations leading to *CDKN2A* and *TP53* loss-of-function. Further genomic alterations, most frequently occurring within *PTEN* and *ARID2A* genes, as well as in the *TERT* promoter region, result in disease progression towards high-grade malignant Spitz melanoma (85, 86). A summary of the main genetic alterations in melanoma is provided in **Table 1**.

**Table 1:** Summary of the main molecular alterations in cutaneous melanoma described in this review.

Main Alterations	Locus	Mutation		Frequency(%)	Pathway	Function
**SOMATIC ACTIVATING MUTATIONS**						
BRAF	7q34	V600E; V600K; K601E		50-70	MAPK signaling	Cell proliferation and survival
NRAS	1p13.2	Q61R/K/L		15-30	MAPK/PI3K signaling	Cell proliferation, differentiation and survival
RAC1	7p22.1	P29S		˜9	MAPK signaling	Cell proliferation and migration
KIT	4q12	L576P; K642E		5-15	MAPK/PI3K and JAK/STAT signaling	Cell proliferation and survival
TERTprom	5p15.33	C228T; C250T		14	Telomerase activity	Cell survival
MAP2K1/MAP2K2	15q22.31/19p13.3	E203K/E207K		˜8	MAPK signaling	Cell proliferation
GNAQ/11	9q21.2/9P13.3	Q209L		rare	MAPK signaling	Cell proliferation
IDH1	2q33.3	R132C/S		˜5	Metabolism of isocitrate	Cell proliferation and impaired differentiation
ERBB2/4	17q21/2q34	L755C; L755S; V777L; P780S; L785F; S341L, R393W		1/19	Tyrosine kinases signaling	Cell proliferation and survival
KRAS	12p12.1	G12V; G12D		˜2	GTPase activity	Cell proliferation and survival
SF3B1	2q33.1	R625C; R625H		33	Alternative splicing	Tumorigenesis
**SOMATIC LOSS-OF-FUNCTION MUTATIONS**						
NF1	17q11.2	C1318T; C3049T; G3497A; C3826T; A4256G; A4267G; C5242T; C5260T; C5380T; T5795C; C5839T (chromosomal aberrations, deletions, insertions, duplications)		10-15	MAPK/PI3K signaling	Cell proliferation, differentiation and survival
PTEN	10q23	A499G; C112T; T416G; G380A; T1032G (deletions, insertions)		14	PI3K signaling	Apoptosis, cell survival and immune evasion
TP53	17p13.1	Several UV-induced		15	Caspase3, FAS and CTL mediated apoptotic pathways	Cell-cycle progression, DNA repair and apoptosis
RASA2	3q23	R310*; S400F		˜5	RAS signaling	Cell proliferation and migration
**GERMLINE LOSS-OF-FUNCTION MUTATIONS**						
CDKN2A	9p21	G101W; E69G		20-40	RB pathway	Apoptosis and cell survival
CDK4	12q14.1	R24H; R24C		NA	G1/S phase cell cycle checkpoint	Cell-cycle progression

*mutation introducing a codon stop that gives rise to a truncated protein.

In addition to genetic variations, increasing evidence supports the involvement of epigenetic modifications, such as gene silencing by non-coding RNAs, in melanoma pathogenesis. Up- and down-regulation of microRNAs (miRNAs) can modulate the expression of target genes governing key signaling pathways responsible for melanoma progression (48, 87, 88).

Although there is still limited data on miRNA expression profiles in melanoma, techniques such as quantitative *in situ* hybridization (qISH) for fluorescent detection of candidate miRNAs, qRT-PCR, *SplintR-qPCR*, and miRNA microarray, have been employed to uncover differential miRNA expression levels in melanomas, in comparison to normal melanocytes and benign melanocytic lesions, as well as between primary and metastatic melanomas (89–91).

The deregulated expression of miRNAs leads to dysregulation of key signaling pathways controlling tumor cell proliferation, cell-to-cell interactions, epithelial-to-mesenchymal transition (EMT) (89, 92, 93), stemness potential (88, 92), as well as senescence (59) and programmed cell death (87, 94), influencing the progression and metastatic process of melanoma. Tumor-suppressor miRNAs, including let-7a/b, miR-23b, -34a/b/c, -132, -137, -191, -192, -194, -200c, -205, -211, -375, -455, -602, -454-3p, -509, and -582, are under-expressed in tumor tissues and melanoma cell lines, while oncogenic miRNAs (oncomiRs) result over-expressed and include miR-10b, -17, -19, -21, -107, -126, -146a, -155, -193b, -214, -221/222, -365, -373, -506–514 cluster, -520c, and -801 (59, 88, 89, 92, 95–97). In primary melanomas, the downregulated expression of several miRNAs, such as miR-125b, -182, -200c, -203, -205, and -211 has been shown, along with increased levels of miR-10b, -221/222 (90, 91). In metastatic specimens, a miRNA expression profile has been proposed consisting of miR-145, -150, -155, -342-3p, -455-3p, and -497, considered predictors of post-recurrence survival (59). The analysis of miRNA expression profile from melanoma lymph node metastases has identified a unique signature consisting of the downregulation of miR-191, combined with the upregulation of miR-193a/b, -338, -365, and let-7, those being predictors of short-term survival in melanoma patients (59).

Melanospheres express high levels of miR-10b, -21, -182-5p, -191-5p, -373, -378d, -520c, -542-3p, -1301, -1915-3p, -3934, -4767, which feasibly control their stemness and metastatic potential (88, 92, 98).

Frequent dysregulation of miRNA expression has been reported in association with the mutational status. Bandarchi and colleagues (96) found that a low expression of miR-193a, -338, and -565 was associated with *BRAF* missense mutations, while a low expression of miR-663 was associated with *NRAS* mutations. However, they did not observe any specific differentially expressed miRNAs between *BRAF-* and *NRAS*-mutated melanomas. Oncogenic BRAF/mitogen-activated protein kinase kinases (MKK)/ERK signaling in melanoma cells modulates a network of miRNAs, by means of downregulation (let-7i, miR-22, -34a/b, -125a, -132, -211) or upregulation (miR-17-5p, -20a, -92b, -106a/b, -221/222) of miRNA expression (99).

High *KIT* gene expression in BRAF^V600K^-mutated melanomas has been reported, concurrent with the significant downregulation of KIT-targeting miRNAs, including miR-222. This suggests that *KIT* and miR-222 might cooperate, by growth and pro-survival signals, toward clinical aggressiveness (32).

*MITF* expression seems to be regulated by miR-26a, -101, -137, -148, -182, -211, -218, -340, and -542-3p. On the other hand, *MITF* transcription factor/oncoprotein modulates miR-146a, -221/222 cluster, and -363 expression levels (59, 100).

## Familial Cutaneous Melanoma

The susceptibility to melanoma is commonly observed in people carrying common variants in lower risk susceptibility genes; however, 5-10% of cases develop in melanoma-prone families, with at least two cases in the same family (101), probably carrying mutations in high penetrance susceptibility genes (102, 103). From an epidemiological perspective, familial melanoma differs from sporadic melanoma for:

- an **earlier age** at diagnosis (104–107)
- a greater proportion of **sunburns**. We could hypothesize that familial cases may have an intrinsic cutaneous reactivity, deriving from some genetic characteristics, such as MC1R or DNA repair capacity (108, 109). However, the high number of sunburns in familial cases demonstrates the absence of carefulness towards the primary prevention
- a higher **number of naevi**, ‘great naevi’ in particular or atypical naevi, especially if on the trunk or the lower limb (110–113). We may hypothesize that the higher number of great or atypical melanocytic naevi depends on sunburns or that it is an independent factor due to genetic pressure, i.e. *CDKN2A* mutation or polymorphisms on chromosome 9 and 22 (114)
- a **more frequent** association of **melanoma on naevus**. Melanomas arise from pre-existent naevi in about 20–30% cases (115). This finding may be the consequence of the presence of a higher number of melanocytic naevi and sunburns in the familial melanoma group, as previously hypothesized
- a greater proportion of multiple primary melanomas (MPMs), in a synchronous or metachronous manner (116)

On the other hand, familial melanoma does not differ from sporadic melanoma with regard to the main histopathological prognostic factors such as Clark’s level and Breslow’s thickness (110). Moreover, in the familial melanomas the diagnostic anticipation is believed to be genetic in nature and not to be due to a better or frequent skin self/medical examination (attributable to increased awareness of the risk). Indeed, *CDKN2A* mutation may represent a biological pressure responsible for the earlier onset of the disease. In particular, germline mutations convey pro-tumorigenic features and often affect the high-risk susceptibility genes *CDKN2A* and, less commonly, *CDK4*, associated with familial melanoma, where the phenotype of *CDKN2A* or *CDK4*-mutated families is indistinguishable (27, 80, 117).

The *CDKN2A* gene is the major high-penetrance familial melanoma predisposition gene, with germline mutations identified in 20%-40% of melanoma families (118). Similarly, *CDKN2A* mutations have been reported associated to MPMs in Italian patients, being more frequent in MPM cases with a positive family history (119).

The tumor suppressor *CDKN2A* is located at the 9p21 locus and encodes 2 different proteins, p16INK4A (p16) and p14ARF (p14), which promote the cell cycle arrest in G1 phase by inhibiting RB protein phosphorylation through CDK4 and act through the p53 pathway inducing cell cycle arrest or favoring apoptosis, respectively (118, 120, 121). Mutations in *CDKN2A* produce an imbalance between functional p16 and Cyclin D1, causing abnormal cell growth. Several recurrent mutations in *CDKN2A* have been described as founder mutations. As an example, glycine (G) to tryptophan (W) mutation at codon 101 (G101W) is considered highly oncogenic since it leads to an impaired interaction with Cyclin Dependent Kinase 4/6 (CDK4/CDK6). Also, the glutamic acid (E) to glycine (G) mutation at codon 69 (E69G) has been reported to be deleterious (27). Variants in *CDKN2A* and other intronic mutations have also been described to predispose to melanoma (122).

The* CDK4* oncogene is the second identified high-penetrance familial melanoma predisposition gene, playing a pivotal role in the G1/S phase cell cycle checkpoint. *CDK4* pathogenetic mutations often arise in codon 24 of exon 2, a critical site for the tumor suppressor protein p16 binding. When *CDK4* is mutated, p16 cannot inhibit the CDK4 kinase activity, resulting in increased phosphorylation of the Retinoblastoma Protein (RB) bound to members of the E2F family of transcription factors, with consequent increased E2F release. E2F activates the transcription of pro-S phase cell cycle genes, promoting G1/S phase transition (118).

In families without mutations in *CDKN2A* and *CDK4* genes, the use of NGS methodologies has allowed the identification of rare germline mutations in a few novel melanoma susceptibility genes, namely BRCA1 Associated Protein 1 *(BAP1), TERT*, Protection of Telomeres 1 (*POT1*), ACD Shelterin Complex Subunit and Telomerase Recruitment Factor (*ACD*), TERF2 Interacting Protein (*TERF2IP*) (high risk genes) and *MC1R, MITF* (low to moderate risk genes).

By investigating a melanoma-prone family by linkage analysis and high-throughput sequencing, disease-segregating germline mutations have been identified in the *TERT* gene, causing up to 2-fold increase in its transcription (123). Telomere maintenance has been uncovered as a crucial pathway in melanoma predisposition. *POT1*, *ACD*, and *TERF2IP* are members of the Shelterin protein complex, crucial for the safeguard of telomeres, and have been also described to be mutated in familial melanoma patients (118, 121).

A summary of the main somatic and germline alterations in melanoma is provided in **Table 1**.

## Genomic Technologies

Over the past decades there have been major advances in our understanding of the human genome, mostly due to the rapid development of genomic technologies that allow the interrogation of hundred-thousand loci and/or provide single base pair resolution. The common denominator of these technologies is the capacity to produce a large amount of data in a number of samples assessed, hence the definition of “high-throughput” technologies. In the biomedical context, the application of high-throughput genomic technologies can be used to identify biological markers (biomarkers) to understand disease course and/or predict treatment response or patient survival (124). Biomarkers can be broadly classified into three categories: diagnostic (for the assessment of presence/absence of disease); predictive (how a patient responds to a treatment) and prognostic (how long a patient survives after intervention) (124). Biomarkers can be assessed at different levels, namely: genome, epigenome and transcriptome. At the DNA (genome) level, high-throughput technologies can be applied to detect Single Nucleotide Variants (SNVs), indels, Structural Variants (SVs), CNVs and fusion genes (125). DNA-sequencing techniques include whole-genome sequencing (WGS, to detect alterations in coding and non-coding regions of the genome), whole-exome sequencing (WES, limited to coding regions) and targeted sequencing (focusing on specific regions of the genome when prior information is available). At the epigenomic level, high-throughput technologies are applied to detect chemical modifications of the DNA which regulate gene expression; both microarray and sequencing technologies can be used to detect and quantify DNA methylation status; chromatin immunoprecipitation sequencing (ChIP-Seq) can be implemented to characterize transcription factor binding sites and patterns of histone modifications (126, 127). At the transcriptome level, high-throughput technologies are applied to study RNA species with mRNA being the most commonly studied form of RNA (128–130).

It is now becoming clear that no two cancers are exactly the same. This concept is leading to the development of individual-specific therapeutic approaches, based on the identification and quantification of specific genomic features (131).

Until now, most of the medical treatments have been the result of the “one-size-fits-all” approach. However, while some treatments can result very effective in some patients, some other patients might not benefit to the same extent or might even have adverse effects from a given therapy. Personalized medicine aims at understanding individual differences in people’s genetic and environmental backgrounds and at giving medical professionals the tools they need to develop tailored and most efficient therapeutic strategies.

It has now been accepted that the integration of the personalized medicine approach into the oncology field may lead to improvement in cancer treatments, especially considering the interindividual variability (131).

With the completion of the Human Genome Project in 2003, scientists have started acquiring the tools to read and interpret individual genetic codes. Since then, technologies have significantly improved. We describe below examples of high-throughput genomic technologies that can be applied to the oncology field. A summary of those technologies is provided in **Table 2**.

**Table 2:** Summary of the advantages and disadvantages of the genomic technologies described in this review.

Technology	Examples	Description	Advantages	Disadvantages
Short-read sequencing	Illumina	Cyclic reversible termination	Cost-effective, overall higher sequence fidelity, supported by several analysis tools	Not able to resolve structural variants, phase alleles and provide coverage for respective regions; GC bias
	SOLiD – Life Technologies	Sequencing by ligation		
	Ion Torrent	Ion semiconductor sequencing		
	Roche/454	Pyrosequencing	First commercial NGS platform; read length up to 1 kb	Inaccuracy in homopolymer sequencing, high error rate, low yield, high cost per bp. Operation has shut down in 2013
Long-read sequencing	Pacific Biosciences	Single Molecule, Real-Time Sequencing	Generate reads in excess of 10 kb; perform *de novo* assembly; mapping certainty; transcript isoform identification; detection of structural variants; direct detection of epigenetic modifications	Lower accuracy per read; bioinformatic challenges including limited pipelines available, coverage biases, overall high error rates
	Oxford Nanopore	Nanopore Sequencing		
Single-cell platforms	Fluidigm C1	Microfluidics-based	Allow the analysis of individual cells; can identify clonal cell subpopulations	Nucleic acid amplification necessary
	Chromium 10X Genomics	Droplet-based		
	BD Rhapsody	Microwell-based		
Spatial genomics	Visium 10X Genomics	Positionally capturing mRNAs from thin tissue sections onto an oligonucleotide array	Resolve genomic information of individual cells within the spatial context of their native tissue	Relatively new
	Nanostring GeoMx DSP	Standard immunofluorescence combined to optical barcoding quantification		
Optical Mapping	Bionano Genomics	High-resolution imaging of long DNA molecules	Resolve complex regions of the genome up to hundreds kbp in length; allow genome finishing	No single bp resolution; specific protocols required for the extraction of DNA of high molecular weight

## Next-Generation Sequencing Technologies

The last decade has witnessed a rapid increase in the number of next-generation sequencing (NGS) technologies implemented, with entire genome sequencing producing gigabases of reads on a daily basis (124, 132–134). The application of NGS technologies is currently providing a more comprehensive understanding of the mutational landscape of cancer and as a consequence, a better understanding of its pathogenesis (20, 21, 135–137).

NGS technologies generally require the conversion of the nucleic acid materials derived from biological specimens into a form that is suitable for sequencing, this step is called “library preparation” and represents perhaps the most challenging step with biological and bioinformatic implications (124, 138). Library preparation is generally characterized by an amplification step by polymerase chain reaction (PCR) (138, 139). This step is particularly prone to bias introduction (138). Although several PCR-free methods are currently available, they are not free of flaws (138, 139). Library preparation methods are of paramount importance when only a small amount of starting material is available and clinical samples cannot be collected again. During the library preparation step, adaptors are ligated to fragmented DNA and then amplified before sequencing. Amplified templates can be generated in solution or on a solid support by covalently attached oligo. On the solid support of the Illumina platform for instance, fragmented adapter-ligated DNA molecules are bound to these primers and amplified through a series of amplifications to generate identical sequences that provide template for the sequencing reaction. Upon library preparation, the sequencing step is performed.

There are different approaches for high-throughput sequencing, according to the genomic platform employed, each of which uses bespoke protocols. Below, we list the most common high-throughput genomic sequencing technologies and provide some examples of their application in the context of melanoma.

The first NGS platform was launched in 2005, and several other methodologies have followed, as reviewed in detail in other reports (132–134, 140). Their major feature is the ability to generate thousands/millions sequence reads at the same time (133, 141).

Illumina is perhaps the most commonly used genomic technology in the research and healthcare settings; the technology employs the so-called flow cell, a solid surface on which adapters are covalently attached; the flow cells adapters are complementary to the library adapters. Illumina uses the principle of cyclic reversible termination where nucleotides chemically modified are used as terminators of the sequencing reaction. In the Illumina sequencing workflow, all four nucleotides are added to each cycle and each of the four nucleotides carries an identifying fluorescent label. Once the right nucleotide gets incorporated, the unincorporated nucleotides are washed away, and the flow cell gets imaged; the fluorescent groups are then chemically cleaved and the 3’-OH groups deblocked to allow the next cycle to occur.

In early 2017, Illumina released the NovaSeq series which exceeded existing sequencing performance metrics and allows multiple applications in the same run (142) (“NovaSeq 6000 System - Illumina: https://www.illumina.com/systems/sequencing-platforms/novaseq.html”) (142).

Studies employing WES and WGS on the Illumina platform have recently improved the characterization of somatic mutations in melanoma and demonstrated that melanoma displays one of the highest rates of somatic mutations as compared to other types of cancers, which makes it challenging to distinguish driver from passenger mutations (24, 143–145). The highest mutation frequency in cutaneous melanoma is explained by the exposure to ultraviolet (UV) radiation, a well-documented carcinogen (143). It was also reported that cutaneous melanoma is particularly prone to cytidine to thymidine transition (C>T). Such alteration is specific of a UV-light induced mutational signature (146).

Another mode of sequencing is represented by the one applied by Life Technologies with the SOLiD (Sequencing by Oligonucleotide Ligation and Detection) NGS system. The chemistry employs a sequencing by ligation method and a template preparation based on the creation of clonal bead populations. DNA fragments are amplified clonally on beads, placed on the solid-phase of a flow cell. In the sequencing by ligation approach, a mix of differently labeled nucleotide probes are flushed into the flow cell. When the correct probe is incorporated, it gets ligated into the primer on the solid-phase; the unincorporated nucleotides are washed away and the fluorescence gets recorded. The fluorescent dye is then removed and the next sequencing cycle commences (147).

A completely different approach to NGS relies on the detection of hydrogen ions released after nucleotide incorporation. This approach was employed by Ion Torrent in 2010, later purchased by Life Technologies and subsequently by Thermo Fisher Scientific. The chips employed in this technology are designed to detect pH changes that occur as the sequencing reaction progresses (148). In a recent study, Manca and colleagues have employed the Ion Torrent PGM (Personal Genome Machine) System to evaluate the mutational concordance between primary and metastatic melanoma (83). The authors showed a high level of concordance in the mutational patterns registered in the primary and metastatic samples, especially with regards to the pathogenic mutations in driver genes (83).

In pyrosequencing such as the sequencing employed by Roche/454, a labeled nucleotide is detected when an inorganic pyrophosphate from the incorporated nucleotide releases a signal following enzymatic transformation (140). Library preparation is performed by random fragmentation of genomic DNA and an emulsion-based PCR. The PCR is employed to clonally amplify template DNA in single droplet-encapsulated reaction beads that contain oligonucleotide probes with complementary sequence to the adaptor binding the DNA fragments. The emulsion PCR beads are attached on a solid surface. The addition of nucleotides complementary to the template strand leads to the production of a chemiluminescent signal recorded by the instrument CCD camera. A specialized software then analyzes the position of the beads and the light flashes with each type of nucleotides that are incorporated into the synthesized DNA (149). The Roche/454 sequencing was the first NGS technology to sequence a complete human genome. The technology has been employed in the diagnostic setting for *BRAF* mutational assessment (150–153). However, the inaccuracy of the technology in homopolymer sequencing, the high error rate, low yield and high cost per bp have largely limited its application. In fact, Roche has shut down the 454-sequencing operation in 2013 as the technology became noncompetitive.

The technologies described above are employed to sequence short reads. Short-read sequencing technologies are cost-effective, accurate and supported by many analysis tools (154). Nevertheless, short reads make it more difficult to reconstruct the original genomic map. Short-read sequencing technologies have additional inherent limitations, including GC bias, difficulties in mapping repetitive elements of the genome, difficulties in discriminating paralogues sequences and in allele-phasing (155).

Newer technologies include Pacific Biosciences (PacBio) and Oxford Nanopore Technologies (ONT), both platforms being employed for the so called “long-read sequencing”. While short-read sequencing technologies produce reads of up to 600 bases, long-read sequencing technologies produce reads in excess of 10 kb (154, 156). Those long-read sequencing technologies have considerable advantages, including longer read lengths, the direct detection of epigenetic modifications, the capability to resolve repetitive elements, to allow the characterization of full-length transcriptomes and to allow variant phasing (155, 157, 158). Long-reads also carry more information about structural variation as compared to short-reads. Long-read sequencing is already considered the gold standard for some applications, as for instance the HLA (Human Leukocyte Antigen) typing for tissue transplants. The long-read sequencing technologies are expected to open up new avenues for melanoma characterization and development of targeted therapeutic strategies.

The technology employed by PacBio interrogates a single molecule of DNA in real time. The technology is characterized by the absence of PCR amplification and by the real-time acquisition of the signal. PacBio launched the Sequel II system in 2019 which by employing the High Fidelity (Hi-Fi) sequencing mode allows for high fidelity reads and a superior call rate when compared to other technologies, as demonstrated by the recent Precision FDA Truth Challenge V2 that evaluated different technologies for variant calling in human genomes and demonstrated a higher performance of the PacBio HiFi technology as compared to Illumina and ONT (159) (“PrecisionFDA Truth Challenge V2: Calling Variants from Short and Long Reads in Difficult-to-Map Regions: https://precision.fda.gov/challenges/10/view/results”) (159). The Single Molecule Real-Time (SMRT) Sequencing employed by PacBio can also be used to detect methylation changes in the genome. The technology relies on the kinetics of polymerase incorporation of individual nucleotides, allowing the direct detection of these modified cytosines (160, 161). The PacBio system was the first to be launched as “third-generation sequencing”. Sequencing occurs into the so-called “zero mode waveguides” (ZMW), that are single pockets where DNA and polymerase bind to and where the signal is detected by the incorporation of phosphate-labeled nucleotides to the well (162). In the latest Sequel II system, the SMRT cells used for sequencing contain 8M ZMW which represents an improvement of the data output as compared to the previous SMRT cells that contain 1M ZMW.

In ONT sequencing single-stranded DNA molecules are driven into nanopores; when each nucleotide of the DNA strands partially obstructs the nanopore, an alteration of the electrical property is recorded and analyzed (163, 164). Since the technology uses unmodified DNA, the major advantage consists into yielding results very quickly from minimal starting quantities. The first prototype of the platform consisted in the MinION that was launched in the market in 2014 (147).

Despite the technical advantages of long-read sequencing technologies, their application in the field of cancer has been very limited. Cavalier and colleagues (165) employed SMRT sequencing for the detection of tyrosine kinase inhibitors (TKI) resistance mutations down to a level of 1% in chronic myeloid leukemia (CML) patients. Additionally, they were able to phase co-existing mutations, providing new information about the clonal distribution of resistance mutations in BCR-ABL1. Other two studies have applied long-read sequencing for the detection of multiple *TP53* mutations distributed in different alleles in acute myeloblastic leukemia (AML) and myelodysplastic syndrome (MDS) and for phasing of somatic mosaicism mutations in GJB2 in a patient with keratitis-ichthyosis-deafness syndrome, respectively (166, 167). Despite the examples above and to the best of our knowledge, the long-read sequencing technologies have not yet been applied to the field of cutaneous melanoma. They could offer many advantages especially with regards to the study of SV, insertions, deletions, duplications, inversions or translocations. SV unfortunately have been neglected from a proper characterization in cutaneous melanoma despite being an important source of diversity between genomes and despite being proved to be relevant in human health (154, 168, 169).

Other advantages of long read-sequencing technologies rely in the possibility to sequence full length transcripts and identify novel splicing isoforms (155, 170) as well as detect base modifications (156). As an example, in SMRT sequencing, base modifications are inferred from the delay between fluorescent pulses, referred to as interpulse duration (IPD) (171). SMRT sequencing allows the detection of 6mA, 4mC, 5mC, and 5hmC DNA modifications, although at different sensitivity (171). In nanopore sequencing, modified RNA or DNA bases affect the flow of the current through the pore differently than non-modified bases, resulting in signal shifts (172).

While the implementation of long-read sequencing technologies on large scale projects is limited by the cost and community expertise, we expect this to change rapidly. We believe that the application of these newer technologies will make it possible to resolve complex regions of the genome and to characterize the epigenome landscape and the full-length transcriptome of cutaneous melanoma. Additionally, the integration of the data produced by short- and long-reads technologies will produce more complete and contiguous genomes, which will open exciting avenues in genomics as well as facilitate the further understanding of the molecular mechanisms underlying melanoma onset and progression. NGS technologies will also provide a useful tool for the development of therapeutic strategies tailored to the genetic makeup of individual cutaneous melanomas.

Another important technology worth to note is represented by single-cell sequencing, which is a powerful approach to explore the organization and function of the tumor microenvironment. Cutaneous melanoma is characterized by tumor heterogeneity, which represents a relevant obstacle for its treatment. The bulk sequencing techniques cannot identify rare clonal subpopulations that might be responsible of tumor aggressiveness or resistance to therapy. The application of single-cell sequencing technologies allows the analysis of DNA sequences, epigenetic markers and gene expression patterns in individual cells (173). Single-cell sequencing technology encompasses the following steps: i. isolation of single cells; ii. isolation and amplification of genetic material; iii. sequencing of the genetic material and data analysis (174). The capture of individual cells can be pursued through micromanipulation, fluorescence-activated cell sorting (FACS), magnetic-activated cell sorting (MACS) and microfluidics (173, 175). Such approaches require cells or nuclei to be in suspension, thus they cannot always maintain the spatial context in tissues. Laser capture microdissection (LCM) bypasses this limitation and can also be used to isolate rare cells. When comparing single-cell DNA sequencing to single-cell RNA sequencing, the first method has been proven more challenging than the latter one (173). DNA amplification is necessary when performing single-cell DNA sequencing. DNA amplification methods mainly include the degenerative oligonucleotide PCR (DOP-PCR), which provides uniform amplification but a low coverage and the multiple displacement amplification (MDA), that uses polymerase strand displacement activity and can lead to a high genome coverage but with a non-uniform amplification. Several alternative methods have been refined to decrease allelic drop-out and false positive rate (176). Those methods include: Nuc-seq, which sorts nuclei in G2/M phase; the multiple annealing and looping-based amplification cycle (MALBAC), which uses quasi-linear preamplification coupled with strand displacement active polymerase; and the micro-well displacement amplification system (MIDAS), that employs small reaction volumes and eliminates non-uniform amplification (177). Once amplified, DNA is provided as substrate for library construction for NGS. So far, Illumina seems to be the most employed platform due to low cost per base at high throughput.

To sequence the transcriptome of a single-cell, RNA undergoes to a whole transcriptome amplification (WTA) step. Initial WTA methods engaged the T7 RNA polymerase for amplifying cDNA linearly though *in vitro* transcription (IVT) (178). Further methods included oligo d(T) primers attached to adaptor sequences for the reverse transcription step and amplification of polyadenylated mRNA by PCR (179). However, these methods are not free of flaws as they display 3’ mRNA bias. To overcome such bias, the SMART-Seq method has been introduced, which amplifies only full-length mRNA transcripts using a reverse transcriptase from the Moloney Murine Leukemia Virus (MMLV), with template-switching and terminal transferase activity (173, 180). The SMART-Seq2 method was further developed and led to an improved detection, coverage and accuracy as compared to SMART-Seq method (181). Additional protocols are also available for transcriptome analysis and include single-cell tagged reverse transcription (STRT), cell expression by linear amplification and sequencing (CEL-seq), CEL-seq2, QuartzSeq, droplet-based RNA-seq, and massively parallel RNA single-cell sequencing (MARS-seq) (173, 182–185). Currently, there exist many commercial platforms for modern-approaches of single-cell sequencing. The Fluidigm C1 is a microfluidics-based system that captures individual cells through integrated fluidic circuits (186). However, the employment of such platform has been limited due to the low throughput and the cell size bias because of its determined size range of the capture site for a given chip (187). The Chromium system from 10X Genomics is a droplet-based platform displaying high sensitivity, high accuracy, low technical noise and high cost (173). Drop-Seq, which is also a droplet-based platform, represents a more cost-efficient solution as compared to the Chromium system. The BD Rhapsody system for single-cell analysis is a microwell-based platform that is used for targeted RNA sequencing, thus more useful when aiming at detecting rare information (188). Additional platforms for single-cell analysis have been described elsewhere (188).

Single-cell sequencing has been employed in melanoma. An interesting study has recently investigated the role of heterogenous spheroids in the stromal niche of cutaneous melanoma by single-cell RNA sequencing (189). The authors identified molecules that could play a role in the control of the interaction between melanoma cells and cancer-associated fibroblasts. Another important study applied single-cell RNA sequencing to assess the transcriptomes of single cells cultured from patients’ biopsies with different BRAF and NRAS mutational profiles. The authors were able to identify sub-populations of cells defined by transcriptional modules involved in proliferation, oxidative phosphorylation, pigmentation and cellular stroma (190). We expect that with the advancement of the genomic technologies, more groups will employ single-cell sequencing to shed light on the molecular mechanisms underlying cutaneous melanoma pathogenesis and responsiveness to therapy.

## Spatial Genomics

The combination of state-of-the-art genomic technologies to high-resolution microscopy has led to the establishment of the so-called spatial genomics, an innovative technology that aims at resolving genomic information of individual cells within the spatial context of their native tissue. The general methodology overlays genomic data on a tissue section to provide spatial context (191). The two major players in the spatial genomic field are represented by the Visium technology from 10X Genomics (192), and the Nanostring GeoMx Digital Spatial Profiler (DSP) (193). The 10X Genomics Visium technology captures mRNA molecules from thin tissue sections initially imaged histologically onto an oligonucleotide array. cDNA is then synthesized from the captured mRNA and used for library preparation. Libraries are finally sequenced, and the data processed to identify transcripts and measure their expression. The Nanostring GeoMx DSP platform provides morphological context with high-plex protein or gene expression profiling. Individual slides are first fluorescently stained to allow the GeoMx platform to capture images with morphological context. The technology relies on the use of photocleavable oligonucleotide tags that are attached to antibodies through a light-sensitive linker. The high-plex oligos then get separated from the antibodies or RNA in the region of interest through UV light. Finally, the photocleaved oligos are retrieved from the surface of the tissue and processed for quantitative analysis. The Nanostring GeoMx platform has been applied to carry high-plex characterization of B- and T-cells in melanoma tumors (194). The study revealed that tertiary lymphoid structures play a crucial role in melanoma immune microenvironment through conferring different T-cell phenotypes, thus suggesting that the formation of tertiary lymphoid structures should be investigated to foster responses to cancer immunotherapy (194).

The application of spatial transcriptomics for the study of cutaneous melanoma has also revealed a complex transcriptional landscape of lymph node metastases in a spatial context (195).

The 10X Genomics Visium technology has been applied to skin squamous cells carcinoma (196). The authors identified multiple cells responsible for immunosuppressive functions in dendritic cells, exhausted T cells and Tregs, refining local tumor structures. Spatial genomics offers a great potential to uncover the mechanisms that govern cell interaction in the tumor microenvironment (197) and we expect this field to expand significantly along with advancement of genomic technologies.

## Optical Mapping Technology

Genomic SVs have been well established to be associated with cancer. Genomic SVs arise from the genome instability created during cancer onset and progression (198). Nevertheless, SV analysis of cancer genomes has been severely limited to date by technical shortcomings. Traditionally, SVs have been detected by microarray (limited to imbalanced copy number variation (CNV) with a short dynamic range, low resolution, and relative readouts), next-generation sequencing (NGS) (primarily CNV, some balanced events but too short to span most repeats) and karyotyping and fluorescence *in situ* hybridization (FISH) (both are very low resolution). The optical mapping technology from Bionano Genomics is able to interrogate genome structural differences of hundreds of kilobase pairs and span interspersed and even long tandem repeats making it ideally suitable for elucidating the structure and copy number of complex regions of the genome, such as complex pseudogene and paralogous gene families. The platform does not produce single base pair resolution as it uses an optical mapping technique. Long molecules of DNA are first isolated using Bionano specific extraction methods (DNA >100kbp), the DNA is labeled at specific motifs through labeling enzymes and linearized through nanochannels for visualization. The Bionano technology can identify megabases-long CNVs as well as long-range translocation and other rearrangements (“Bionano Genomics: https://bionanogenomics.com”) (199). An interesting study from Xu and colleagues applied optical mapping technology to study leukemia SVs. The authors identified new SVs in leukemia samples and underscored that the missed knowledge of SVs in cancer samples might hamper advancement in the development of diagnostic and therapeutic strategies (200). By combining WGS to optical mapping, they were able to recover twice as many SVs as revealed by WGS alone. Additionally, they were able to pinpoint variants that likely arose as somatic alterations.

To the best of our knowledge, the optical mapping technology has not yet been used for the investigation of SVs in cutaneous melanoma and its application may lead to useful insights for cutaneous melanoma characterization and to a better clinical management.

## International Efforts

The increase of whole genome sequencing and transcriptome sequencing data following the implementation of NGS technologies offered the possibility to perform meta-analysis studies aiming at identifying patterns of genomic alterations across different tumor types (201). Several consortia were established with the aim to federate a large amount of sequencing data of cancer genomes.

The International Cancer Genome Consortium (ICGC) was first established in 2007 to study the genomes of ~25,000 primary untreated cancers as part of the “25K Initiative” (202) (“The International Cancer Genome Consortium: https://icgc.org”) (202). In a later phase, the ICGC launched the Pan Cancer Analysis of Whole Genomes (PCAWG), also known as the Pan-Cancer Project. A technical working group was assembled to develop the informatic pipelines by aggregating the raw data from different groups that studied individual tumor types and by aligning the sequences to the human genome. This made it possible to generate a set of high-quality somatic mutation calls for the downstream analyses (201–203). ICGC has also planned another initiative, named “The ARGO (Accelerate Research in Genomic Oncology) Project” aiming at using clinical questions and patient clinical data to drive the interrogation of cancer genomes. The ARGO Project is expected to provide a unique resource of multi-omics data for cancer patients undergoing clinical trials in order to facilitate the discovery of new therapeutic strategies. As of October, 2020 the ICGC repository includes two skin cancer and one melanoma projects (**Supplementary Figure 1**).

The “Catalogue Of Somatic Mutations In Cancer” (COSMIC) represents an additional resource to explore the impact of somatic mutations in cancer. The COSMIC database was launched in 2004 with data from just four genes (204). The resource continued to expand rapidly and by 2005 it included 529 genes from more than hundred thousand tumors (204). A new version of the resource has been launched on August 27, 2020 and it includes 1,459,483 samples. It encompasses a curated update on spliceosomes and also the launch of a new product, “The Cancer Mutation Census (CSM)” (205) (“COSMIC, The Catalogue Of Somatic Mutations In Cancer: https://cancer.sanger.ac.uk/cosmic”) (205).

The Cancer Genome Atlas (TCGA) represents another initiative empowering cancer genome data analysis to facilitate our understanding of the molecular mechanisms underlying cancer development. The project began in 2006 when it was launched as a three years pilot project with a conjunct investment from the National Cancer Institute (NCI) and the National Human Genome Research Institute (NHGRI) (206) [“The Cancer Genome Atlas (TCGA): https://www.cancer.gov/about-nci/organization/ccg/research/structural-genomics/tcga”] (206). The project has characterized over 20,000 primary cancer and matched normal samples encompassing 33 cancer types (206) [“The Cancer Genome Atlas (TCGA): https://www.cancer.gov/about-nci/organization/ccg/research/structural-genomics/tcga”] (206). The TCGA has generated petabytes of genomic, epigenomic, transcriptomic and proteomic data; such data is publicly available and has already led to improvements in the diagnosis, treatment and prevention of cancers. The TGCA repository includes data from 470 characterized cases of cutaneous melanoma, of which 331 samples have been employed in an integrative analysis that included WGS, WES and RNA-sequencing. Such integrative analysis aimed at establishing a framework for the cutaneous melanoma classification into four subtypes that can help clinicians in making decisions for targeted therapies (207). Those four subtypes included: i. the BRAF subtype which accounts for the majority of cutaneous melanomas (~52%) and it is characterized by the presence of a mutation on the *BRAF* gene; ii. the RAS subtype defined by the presence of mutations on the *RAS* gene, accounting for ~28% of cutaneous melanomas; iii. the NF1 subtype characterized by the presence of mutation on *NF1* gene and accounting for ~14% of cutaneous melanomas; iv. the Triple Wild-Type subtype, a more heterogenous subgroup characterized by the absence of mutations on *BRAF*, *RAS* and *NF1* genes. The study reported some interesting findings, including that the patients in the BRAF subtype were younger than the patients in the other groups, while the opposite was observed for patients in the NF1 group. The Triple Wild-Type subtype showed a significant higher number of copy-number segments and displayed more focal amplifications including known oncogenes as compared to the other groups. The same study also showed that a subset of each of the genomic classes of cutaneous melanoma expressed markers indicative of immune infiltration that were associated with improved survival and could carry clinical relevance for immunotherapy treatments (207).

Another useful tool for Cancer Genomics is cBioPortal which provides visualization, analysis and download of large-scale cancer genomics data sets (208) (“cBioPortal: https://www.cbioportal.org”) (208). The Portal was initially developed at Memorial Sloan Kettering Cancer Center (MSK) and the cBioPortal software is now available under an open-source license *via* GitHub. The maintenance of the software is performed by a multi-institutional team that includes MSK, the Dana Farber Cancer Institute, the Princess Margaret Cancer Centre in Toronto, the Children’s Hospital of Philadelphia, The Hyve in the Netherlands and Bilkent University in Ankara, Turkey (208) (“cBioPortal: https://www.cbioportal.org”) (208). The advantage of cBioPortal relies on the user-friendly interface, an example of the data retrieved from cBioPortal is displayed in **Supplementary Figure 2**. The interface shows graphs from 471 patients.

Another important consortium worth of mentioning is the GenoMEL, the Melanoma Genetics Consortium, that represents a non-profit consortium launched in 1997 that includes research groups worldwide and it is focused on the study of genetics in familial melanoma (“GenoMEL, the Melanoma Genetics Consortium: https://genomel.org/research/programme-and-aims/”) (209).

Additionally, a unique collaboration of multidisciplinary experts from the European Dermatology Forum (EDF), the European Association of Dermato-Oncology (EADO), and the European Organization of Research and Treatment of Cancer (EORTC) was formed to make recommendations on cutaneous melanoma diagnosis and treatment, based on systematic literature reviews and the experts’ experience (5).

The combined efforts of international consortia described above has the potential to provide new insights into the genetic makeup of cutaneous melanoma as well as identifying novel molecular defects that can improve our understanding of cutaneous melanoma pathogenesis.

## How Genomic Technologies Are Moving Toward Personalized Medicine

Cutaneous melanoma, especially in metastatic stage, represents a challenging clinical situation with a steady need for effective treatment options. The past and current findings on the mutational profile of cutaneous melanoma are opening new doors to understand how this tumor initiates, progresses and metastasizes and are leading to a new orientation for antitumor therapy, referred as targeted therapy, which offers the opportunity for various treatment options that can be used in combination with other treatment modalities, i.e., surgical resection, chemotherapy, radiotherapy, and immunotherapy.

The dramatic importance of molecular biology-based strategies used for the detection of driving mutations in melanoma oncogenes resides in defining targetable alterations and making them “druggable”, thus enabling meaningful advances in personalized medicine (210–212).

Since the first step to an efficient therapy is to identify which patients will derive most benefit from given treatments, a growing number of translational studies is now focused on the identification of biomarkers useful in the selection of patients eligible for specific treatments (213, 214). The critical role of MAPK/ERK signaling pathway in melanoma has been used for the development of targeted treatments. Since the activation of MAPK/ERK signaling is often due to mutations in the *BRAF* and *NRAS* genes, mutation testing for these genes has become a standard procedure to guide the oncologist’s therapeutic choice and predict the course of therapy (215–217). For instance, only patients with a BRAF^V600E^-mutated melanoma are expected to benefit from targeted therapies with BRAF/mitogen-activated protein kinase (MEK) inhibitors, while patients with a BRAF^K601E^-positive melanoma respond only to a minority of those drugs, such as trametinib (33). Moreover, recent studies have indicated that BRAF^V600E^ detection through circulating tumor DNA prior to treatment is predictive of response to BRAF/MEK inhibitors (218). Recently, there have been major advancements in the treatment of cutaneous melanoma, due to the introduction of targeted therapies, including for example vemurafenib and dabrafenib (BRAF kinase inhibitors) and trametinib and cobimetinib (MEK inhibitors) (219–221).

As another example, the preclinical observation that CDK4/6 inhibition can attenuate *NRAS* oncogenic signaling when combined with MEK inhibition has led to an undergoing clinical investigation of the synergistic inhibition of CDK4/6 (PD-0332991) and MEK1/2 (selumetinib) in NRAS-mutant melanomas (29).

Despite the advances in the development of novel antitumor approaches, resistance to targeted therapy is a noteworthy issue in the management of melanoma patients, being driven by multiple mechanisms. High genomic instability and heterogeneity can promote primary (*de novo*) or acquired resistance (occurring in tumors previously responsive to the same treatment) (222, 223). A lack of treatment response and poorer progression-free survival have been observed in patients with BRAFV600-mutated metastatic melanoma, treated by MAPK inhibitors, and with coexisting genetic alterations, such as the *TERT*prom c-146C>T mutation, which can affect the MAPK pathway blockade (37). Other mechanisms responsible for MAPK reactivation and sustained ERK signaling include alterations in *MEK* and *NF1* genes. Additionally, the overexpression of the RAF isoform, Raf-1 Proto-Oncogene, Serine/Threonine Kinase (*CRAF*), can induce resistance to BRAF inhibitors by MEK activation or by paradoxical transactivation of RAF dimers, promoting ERK signaling (224, 225). Similarly, poor response to BRAF inhibitors in patients with BRAF-mutant melanoma has been correlated to concurrent loss-of-function mutations in the *PTEN* gene, which can lead to the reactivation of the PI3K/AKT pathway (226). MAPK and PI3K/AKT pathways have also been reported to get reactivated by the expression of miR-204-5p and miR-211-5p in response to short-term treatment with BRAF inhibitors (224).

Co-targeting signaling effectors downstream of driver oncogenes represents an actionable strategy to overcome resistance to BRAF inhibitors. MEK is a downstream effector of BRAF. The combination of targeted therapy with BRAF/MEK inhibitors is being applied routinely in the clinic, significantly improving the response rates of patients with BRAF-mutant metastatic melanoma (227–229). The combination of BRAF and/or MEK inhibitors with immune checkpoint inhibitors is a further option in clinical practice. Since the activation of the Programmed Cell Death Protein 1 (PD-1)/Programmed Death-Ligand 1/2 (PDL-1/2) axis is often exploited by tumor cells to escape immune-mediated death, the use of anti-PD-1 or anti-PDL-1 monoclonal antibodies, in combination with BRAF/MEK inhibitors, has been proven to improve therapeutic response and progression-free survival of cutaneous melanoma patients (223, 230, 231). Recently, it has also been suggested that *TP53* mutation leads to downregulated FAS levels, which impede the induction of apoptosis, limiting the response to immune checkpoint inhibitors, such as anti-cytotoxic T-lymphocyte antigen-4 (CTLA-4), thus serving as a negative predictor of response to therapy (61, 231). *PTEN* silencing in BRAF-mutant melanoma cell lines has been associated to a decreased ability of T-cells to kill the tumors (57).

Our understanding on tumor biology is now allowing testing patients for a broader number of genes at the same time (232). NGS technologies are able to identify genetic aberrations, including rearrangements, CNVs, insertion, and deletions, that have been previously neglected from the clinical testing. NGS-based multigene panels offer a targeted method to assess several genes simultaneously (233). These tests have also the capability to identify specific actionable driver mutations and help in understanding the underlying mechanisms of drug resistance to point out patients more likely to respond to a given therapy. An interesting study from Diefenbach and colleagues has proposed a melanoma NGS multigene panel for the analysis of circulating tumor DNA (234). The panel included 123 amplicons in 30 genes encompassing targetable mutations as well as alterations associated with resistance to treatment. Such panel represented an improvement to the UltraSEEK Melanoma Panel from Agena Bioscience, which can detect 55 clinically relevant variants across 13 genes (235). Another example of melanoma multigene NGS panel is represented by the VarMap NGS panel which includes 8 genes frequently mutated in melanoma and employs the NuProbe’s PCR based quantitative Blocker Displacement Amplification (qBDA) technology (236) to allow detection of variants at low frequency. Other NGS-based panel for melanoma include the OnkoSight panel (237), the NeoTYPE panel (238) and the SureSeq myPanel (239) among others.

NGS has also been applied to identify potentially actionable DNA alterations that could explain resistance to targeted therapy. An interesting study has identified resistance-related mutations in BRAF positive patients that initially achieved partial or complete response to BRAF inhibitors but whose melanoma later progressed (240).

While the value of NGS for the identification of driver/actionable mutations in cutaneous melanoma is being recognized, scientists have started appreciating also the role of melanoma high mutational load attributed to UV mutagenesis (38). Cutaneous melanoma has been shown to exhibit a high tumor mutational burden (TMB), defined as the total number of somatic mutations per million bases, as compared to other tumors (146, 241). The high TMB has been attributed to C>T transitions induced by UV light and makes cutaneous melanoma highly immunogenic (242), thus most suitable for immunotherapy. In fact, the TMB in melanoma has been shown to associate to immune infiltration, response to immunotherapy and prognosis (241).

As genomic technologies continue to evolve, we might see a switch from a targeted approach to a genome-wide approach to study melanoma. A refined molecular classification of cutaneous melanomas by high-throughput genomic technologies has the potential to lead toward a more rational approach to therapy, including patient stratification in subgroups that are genetically more homogeneous and likely to differ in clinical variables, including the pattern of metastasis, disease outcome, clinical response to therapy, thus aiming at personalized treatment approaches (227, 243).

The improved genomic characterization of cutaneous melanoma represents a critical asset with diagnostic and prognostic implications, helping the dermatopathologists in the challenging classification of melanocytic lesions as benign, intermediate, or malignant (48, 84). Defining a mutational signature of driver mutations can also help in identifying those lesions more likely to progress toward high grade melanoma (81).

As a matter of fact, in the era of targeted therapies, molecular subtyping of melanoma is replacing the traditional clinicopathological classification. As an example, based on exome and genome sequencing studies, the TCGA Network has classified cutaneous melanoma into four distinct molecular subtypes: BRAF-mutant, NRAS-mutant, NF1-mutant, and BRAF/NRAS/NF1 wild-type (triple-wild-type group), as described above.

Knowing the tumor genetic signature would be helpful also in the retrospective analysis of clinical trials’ data (243).

Finally, gene mutational status analysis could be also helpful as predictor of response to immunotherapy, a novel approach that has revolutionized the management of metastatic melanoma (51, 244, 245).

## Conclusion

The application of high-throughput technologies holds the promise of personalized medicine, refining the current classification of cutaneous melanoma and allowing the employment of sequencing tests that can guide patient management decisions. Personalized medicine also aims at avoiding the use of potentially harmful treatment strategies, like chemotherapy for instance, by establishing where those treatments are not beneficial for given patients (246).

While the employment of genetic testing in the clinical management of cutaneous melanoma is very well documented (215, 247–250), the application of genomic profiling through high-throughput technologies in the treatment of melanoma is still in its infancy.

NGS technologies are not limitations free. In fact, when applied alone, they cannot capture the entire complexity of melanoma biology. Additionally, not all the newly released genomic technologies have been applied to the study of melanoma. The cost of sequencing technologies is also an important limitation. We expect that with the advancement in sequencing technologies and with the drop in prices, the field of cutaneous melanoma will benefit from new discoveries and these technologies will allow an improved treatment of cutaneous melanoma patients.

We hope that this review provides an up-to-date overview of genomic technologies in the context of melanoma classification and eventually facilitates the application of personalized medicine.

## Author Contributions

This work was conceived and planned by ST. The original draft preparation and writing were made by CS, DM, and ST. All authors contributed to the article and approved the submitted version.

## Conflict of Interest

The authors declare that the research was conducted in the absence of any commercial or financial relationships that could be construed as a potential conflict of interest.

## Glossary

**Table:**

ACD	ACD Shelterin Complex Subunit and Telomerase Recruitment Factor
AKT	AKT Serine/Threonine Kinase
ALK	Anaplastic Lymphoma Receptor Tyrosine Kinase
ALM	acral lentiginous melanoma
AM	acral melanoma
AML	Acute Myeloblastic Leukemia
ARF	Alternate Reading Frame
ARGO	Accelerate Research in Genomic Oncology
ARID2	AT-rich interaction domain 2
AST	atypical Spitz tumor
ATM	Ataxia telangiectasia mutated
ATR	ataxia telangiectasia and Rad3-related
BAP1	BRCA1 Associated Protein 1
BRAF	B-Raf Proto-Oncogene Serine/Threonine Kinase
CDK4/6	Cyclin-Dependent Kinase 4/6CDKN2A: Cyclin-Dependent Kinase Inhibitor 2A
ChiP-Seq	Chromatin Immunoprecipitation Sequencing
CHK	Checkpoint kinase
CML	Chronic Myeloid Leukemia
CNV	Copy Number Variation
COSMIC	Catalogue of Somatic Mutations in Cancer
CRAF	Raf-1 Proto-Oncogene Serine/Threonine Kinase
CSD	cumulative sun damage
CSM	Cancer Mutation Census
CTL	Cytotoxic T-Cell
CTLA-4	Cytotoxic T-Lymphocyte Antigen-4
DOP-PCR	degenerative oligonucleotide PCR
DSP	Digital Spatial Profiler
EADO	European Association of Dermato-Oncology
EDF	European Dermatology Forum
EGFR	Epidermal Growth Factor Receptor
EMT	epithelial-to-mesenchymal transition
EORTC	European Organization of Research and Treatment of Cancer
ERBB2/4	Erb-b2 Receptor Tyrosine Kinase 2/4
ERK	Extracellular Signal-Regulated Kinase
FACS	Fluorescence-Activated Cell Sorting
FAS	Fas Cell Surface Death Receptor
FDA	Food and Drug Administration
FISH	Fluorescence In Situ Hybridization
GAP	GTPase-Activating Protein
GDP	Guanosine Diphosphate
GenoMEL	Melanoma Genetics Consortium
GNA11	G-Protein Subunit α11
GNAQ	G Protein Subunit Alpha Q
GOF	gain-of-function
GPCR	G protein-coupled receptors
GRB2	Growth factor receptor-bound protein 2
GTP	Guanosine Triphosphate
Hi-Fi	High Fidelity
HLA	Human Leukocyte Antigen
HRAS	Harvey Rat Sarcoma Viral Oncogene Homolog
IDH1	Isocitrate Dehydrogenase 1
ICGC	International Cancer Genome Consortium
IL-2	Interleukin 2
IPD	Interpulse Duration
IVT	in vitro transcription
JAK	Janus Kinase
KIT	Mast/Stem Cell Growth Factor Receptor Kit
KRAS	Kirsten Rat Sarcoma Viral Oncogene Homolog
LCM	Laser Capture Microdissection
LMM	Lentigo Maligna Melanoma
LOF	Loss-of-Function
MACS	Magnetic-Activated Cell Sorting
MALBAC	Multiple Annealing and Looping-Based Amplification Cycles
MAPK	Mitogen-Activated Protein Kinase
MAP2K1/2	Mitogen-Activated Protein Kinase Kinase ½
MC1R	Melanocortin-1 Receptor
MDA	multiple displacement amplification
MDM2	Mouse Double Minute 2
MDS	Myelodysplastic Syndrome
MEK	mitogen-activated protein kinase kinase
MET RTK	Met Receptor Tyrosine Kinase
MHC	Major Histocompatibility Complex
MIDAS	Micro-well Displacement Amplification System
MITF	Melanocyte Inducing Transcription Factor
MKK	Mitogen-Activated Protein Kinase Kinases
MMLV	Moloney Murine Leukemia Virus
MPM	multiple primary melanoma
MSK	Memorial Sloan Kettering
MST	malignant Spitz tumor
mTOR	mammalian target of rapamycin
NCI	National Cancer Institute
NF1	Neurofibromin 1
NGS	Next-Generation Sequencing
NHGRI	National Human Genome Research Institute
NM	nodular melanoma
NRAS	Neuroblastoma RAS Viral Oncogene Homolog
NTRK1	Neurotrophic Receptor Tyrosine Kinase 1
ONT	Oxford Nanopore Technologies
PCAWG	Pan Cancer Analysis of Whole Genomes
PCR	Polymerase Chain Reaction
PGM	Personal Genome Machine
PI3K	Phosphoinositide-3-Kinase
PKB	Protein Kinase B
POT1	Protection of Telomeres 1
PPP6C	Protein Phosphatase 6 Catalytic Subunit
PTEN	Phosphatase and Tensin Homolog
qBDA	quantitative Blocker Displacement Amplification
qISH	quantitative in situ hybridization
RAC1	Ras-related C3 Botulinum Toxin Substrate 1
RAF	Rapidly Accelerated Fibrosarcoma
RASA2	RAS P21 Protein Activator 2
RB	Retinoblastoma Protein
RET	Ret Proto-Oncogene
ROS1	ROS Proto-Oncogene 1, Receptor Tyrosine Kinase
SF3B1	Splicing Factor 3b Subunit 1
SMRT	Single Molecule Real-Time
SNF	Sucrose Non-Fermentable
SNP	Single Nucleotide Polymorphism
SNV	Single Nucleotide VariantSOLiD Sequencing by Oligonucleotide Ligation and Detection
SOS	Salt Overly Sensitive
SSM	superficial spreading melanoma
STAT	Signal Transducers and Activators of Transcription
STRT	single-cell tagged reverse transcription
SV	Structural Variations
SWI	SWItch
TCGA	The Cancer Genome Atlas
TERF2IP	TERF2 Interacting Protein
TERT	Telomerase Reverse Transcriptase
TERTprom	TERT promoter region
TKI	Tyrosine Kinase Inhibitor
TMB	Tumor Mutational Burden
TP53	Tumor Protein 53
UV	Ultraviolet
WGS	Whole Genome Sequencing
WES	Whole Exome Sequencing
ZMW	Zero Mode Waveguides
