# Supplementary material for: Cutaneous Melanoma Classification: The Importance of High-Throughput Genomic Technologies
Source: Front Oncol. 2021 May 28;11:635488. doi: 10.3389/fonc.2021.635488 (PMC8193952; doi:10.3389/fonc.2021.635488)
Supplement: Supplementary file 1 [file Presentation_1.pdf]

Supplementary Figure 1

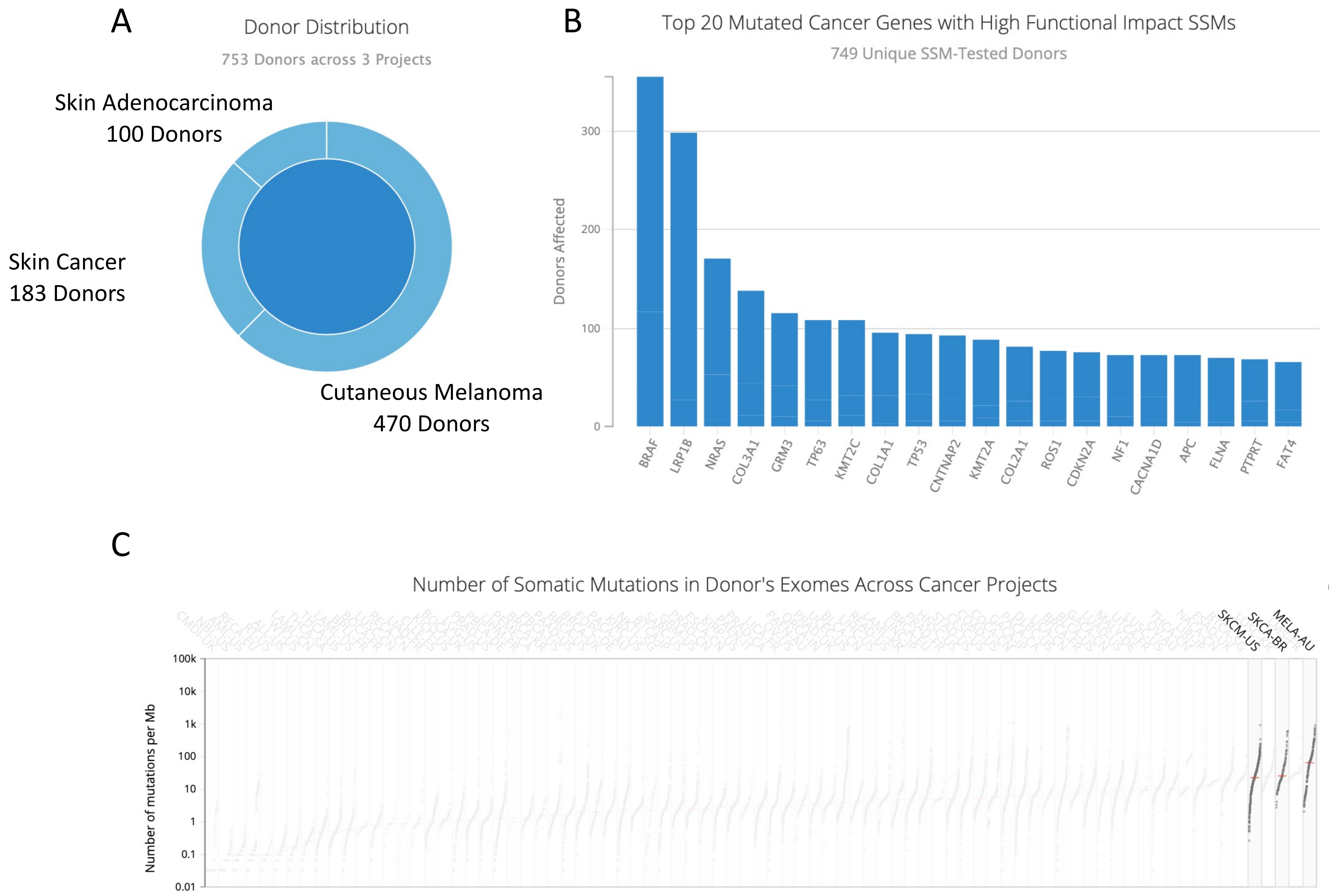

Suppl. Fig. 1. Example of ICGC dashboard. Data breakdown of the two skin cancer and one melanoma projects retrieved from ICGC website (https://icgc.org), as of October 2020.

Supplementary Figure 2

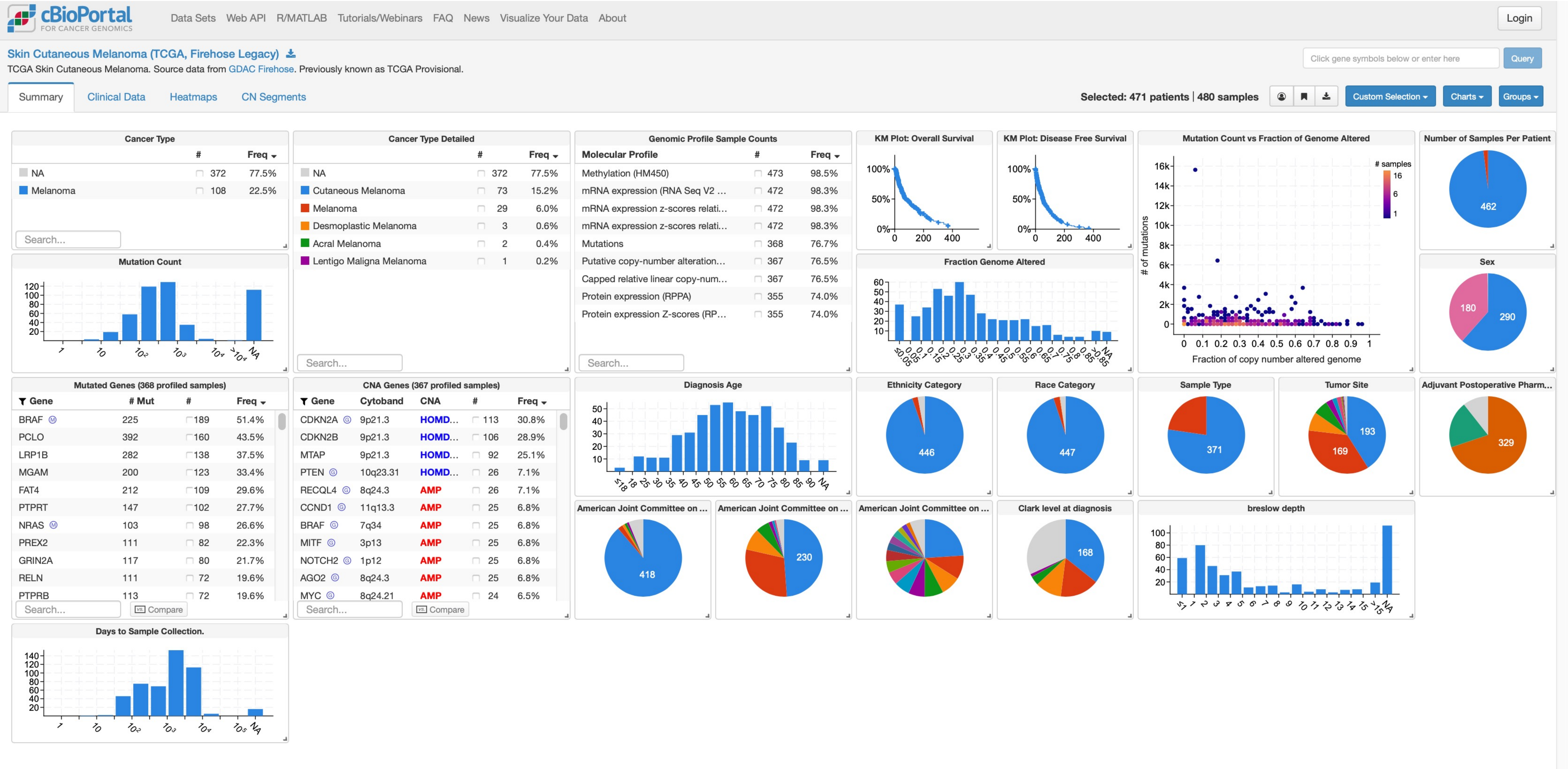

Suppl. Fig. 2. Interface of cBioPortal for 471 patients of skin cutaneous melanoma, as of Oct 2020. Different samples features are displayed by the graphs.
